# Right-sided double valve replacement in an adult patient who underwent surgery for pulmonary stenosis in childhood: a case report

**DOI:** 10.1186/s13019-020-01207-x

**Published:** 2020-07-14

**Authors:** Kimihiro Kobayashi, Tetsuro Uchida, Yoshinori Kuroda, Atsushi Yamashita, Eiichi Ohba, Shingo Nakai, Tomonori Ochiai, Mitsuaki Sadahiro

**Affiliations:** Second Department of Surgery, Faculty of Medicine, Yamagata University, 2-2-2 Iida-Nishi, Yamagata, 990-9585 Japan

**Keywords:** Bioprosthesis, Functional tricuspid regurgitation, Pulmonary valve replacement, Tricuspid valve replacement, Case report

## Abstract

**Background:**

Pulmonary regurgitation and the subsequent functional tricuspid regurgitation are frequently observed in adult patients who previously underwent pulmonary valvular operations. Pulmonary valve replacement, in combination with tricuspid annuloplasty, is frequently performed in adult patients. However, postoperative worsening or recurrence of tricuspid regurgitation is a major concern after pulmonary valve replacement with tricuspid annuloplasty.

**Case presentation:**

Herein, we describe the case of a 56-year-old woman with severe pulmonary regurgitation and functional tricuspid regurgitation after congenital pulmonary stenosis surgery in childhood. Functional tricuspid regurgitation was due to tricuspid annular dilatation, marked right ventricle enlargement, and significant tethering. We performed a bioprosthetic double valve replacement, and the postoperative course was uneventful. The patient is doing well one year after the surgery without prosthetic valve dysfunction.

**Conclusions:**

When functional tricuspid regurgitation is severe and is associated with right ventricular dilatation and subsequent tethering, tricuspid valve replacement rather than annuloplasty should be considered.

## Background

Pulmonary regurgitation (PR) and subsequent functional tricuspid regurgitation (TR) are frequently observed in adult patients who previously underwent definitive repair of congenital right heart diseases, such as pulmonary valve stenosis (PS) and tetralogy of Fallot (TOF) [1]. Although pulmonary valve replacement (PVR), in combination with tricuspid annuloplasty (TAP), is frequently performed, replacement of both the pulmonary and tricuspid valves is rare in this setting. However, when TR is severe and is associated with right ventricular dilatation and subsequent tethering, tricuspid valve replacement (TVR) rather than TAP should be considered. Herein, we present a rare case of both the pulmonary and tricuspid valves being replaced in an adult patient diagnosed with severe PR and TR after PS surgery in childhood.

## Case presentation

**Fig. 1:**
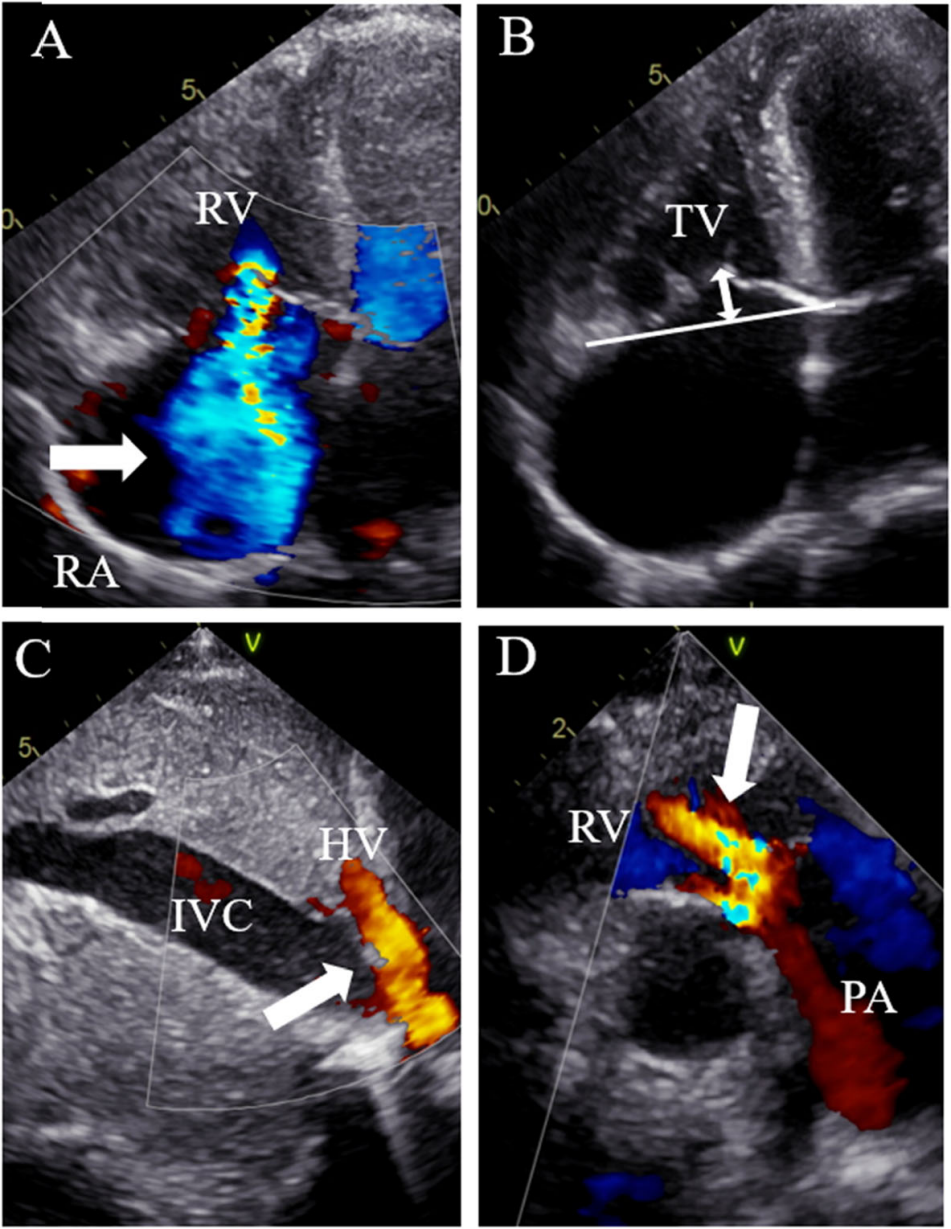
Preoperative transthoracic echocardiography. **a**: Massive tricuspid regurgitation with wide vena contracta. **b**: Extensive tethering height (double headed arrow). **c**: Systolic hepatic vein flow reversal. **d**: Moderate pulmonary regurgitation. RV, right ventricle; RA, right atrium; TV, tricuspid valve; IVC, inferior vena cava; HV, hepatic vein; PA, pulmonary artery

A 56-year-old woman diagnosed with severe PR and TR was referred to our institution. She had previously received surgical relief of congenital pulmonary valvular stenosis at the age of five years. On admission, she was suffering from shortness of breath. A systolic murmur was noted at the left sternal border in the third left intercostal space, and abdominal physical examination revealed four fingerbreadths of hepatomegaly. An electrocardiogram showed atrial fibrillation with bradycardia and low-voltage f wave. Moreover, transthoracic echocardiography demonstrated severe PR and severe TR, and significant amount of TR was owing to the tricuspid annular dilatation (41 mm) and marked right ventricle (RV) enlargement (end-diastolic dimension, 41 mm) with a TR gradient of 26.9 mmHg. The tethering height of the tricuspid valve was 12 mm (Fig. 1). Additionally, the left ventricular dimensions and function were normal, the inferior vena cava was not respiratory-collapsed, and a systolic hepatic vein flow reversal was noted. Cardiac catheterization showed a mild elevation of systolic RV pressure (37 mmHg) with normal mean pulmonary artery pressure (16 mmHg). Although RV dilation (RV end-diastolic volume index 141 ml/m^2^) was revealed by cardiac magnetic resonance imaging (CMR), RV ejection fraction was normal (71%) and tricuspid annular plane systolic excursion (TAPSE) by echocardiography was within normal limits (28 mm), suggesting that RV function was preserved. Liver congestion was demonstrated in both abdominal echography and computed tomography. The patient was judged an acceptable candidate for surgical treatment for severe PR, severe TR, and bradycardia. We did not apply ablative surgical therapy for the atrial fibrillation because of the low-voltage f wave, which is a risk factor for surgical ablation failure.

**Fig. 2:**
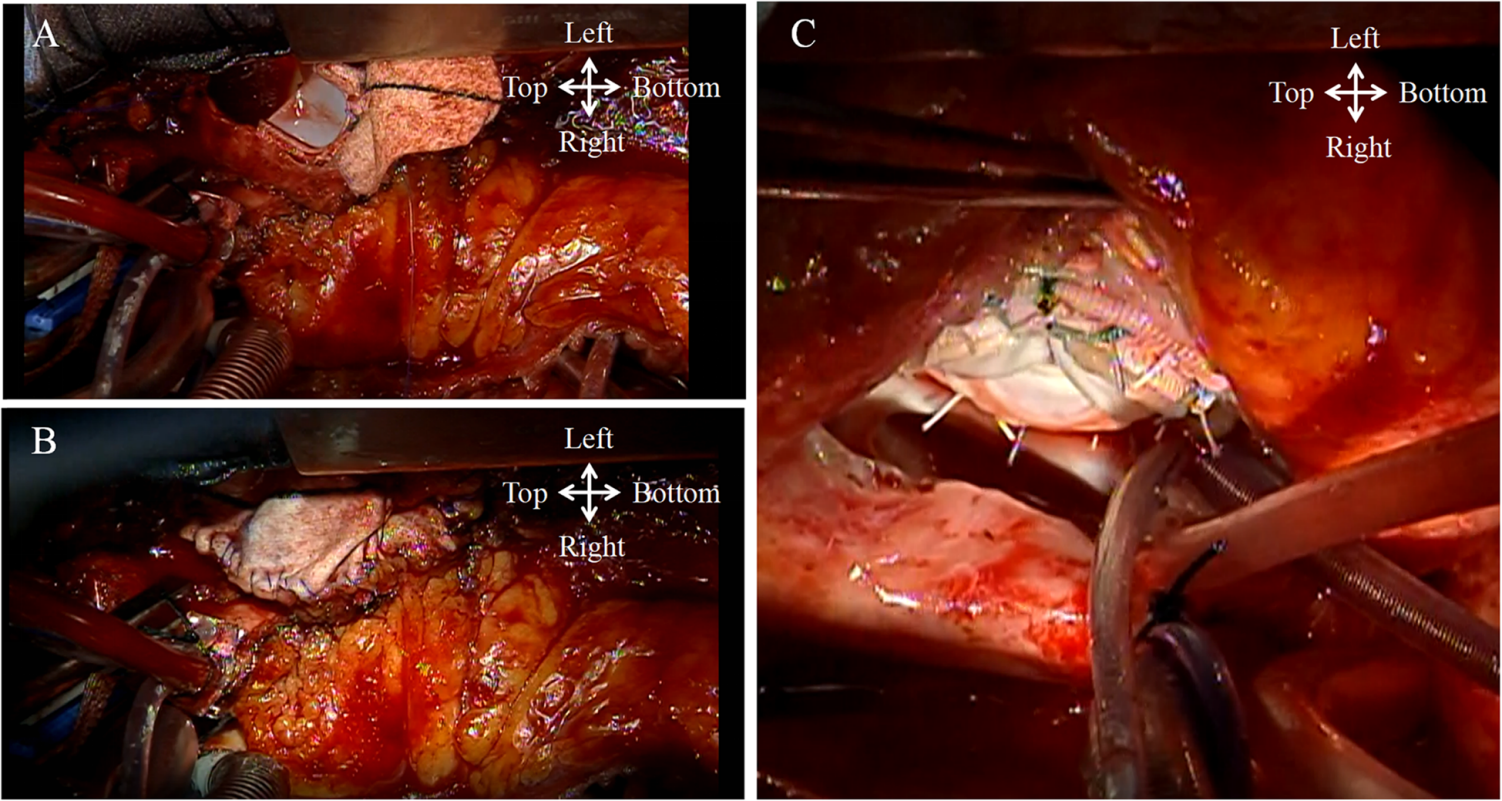
Intraoperative images. **a**, **b**: Pulmonary valve replaced using a bioprosthesis, with reconstruction of the right ventricular outflow tract using a transannular Hemashield patch owing to the small annulus. **c**: Tricuspid valve replaced using a bioprosthesis

Following a sternal re-entry, a cardiopulmonary bypass was established with ascending aortic and bicaval cannulation. The right atrium and ventricle were significantly dilated. After cardioplegic arrest, the pulmonary valve was inspected via a longitudinal incision of the main pulmonary artery. The pulmonary valve was tricuspid but restrictive, which led to valvular incompetence. Furthermore, the pulmonary annulus was so small that an appropriately sized bioprosthesis could not pass through the annulus. Therefore, the incision was extended inferiorly beyond the annulus, and the right ventricular outflow tract was reconstructed using a transannular patch (Fig. 2), followed by PVR with a 19-mm bioprosthesis (Inspiris Resilia, Edwards Life Sciences, Inc., Irvine, CA).

The tricuspid valve was inspected through a right atriotomy. The annulus was significantly enlarged. The valvular tissues were considered fundamentally inadequate for valvular competence even after the undersized annuloplasty. The tricuspid valvular tethering due to right ventricular dilatation was also recognized. We implanted a 27-mm bioprosthetic valve (Epic, St Jude Medical, Inc., St Paul, MN) leaving the leaflet tissue. The epicardial pacemaker lead was subsequently implanted, and a pulse generator was placed in the left rectus abdominis muscle. Weaning from the cardiopulmonary bypass was uneventful with ventricular pacing. The patient showed good postoperative recovery and is currently doing well one year after the surgery, without prosthetic valve dysfunction.

## Discussion and conclusions

Postoperative PR and subsequent functional TR are commonly observed in adult patients who previously underwent surgical treatment for pulmonary valvular disease, such as PS and TOF [1]. Prosthetic valve replacement of the pulmonary valve is frequently required as a definitive repair in this particular pathology. Solitary PVR is considered to lead to improvement of functional TR, therefore, TAP is performed as an adjunctive procedure in patients presenting with TR with annular dilatation [2]. However, postoperative worsening or recurrence of TR is highlighted after PVR and TAP in adult patients who underwent TOF repair in childhood [2]. Similarly, some patients showed recurrent TR and required a redo tricuspid valve surgery after mitral valve surgery and concomitant TAP. In patients with such recurrent TR, a redo TAP or TVR should be considered. However, tricuspid valve reoperations are associated with considerably high operative mortality and morbidity [3].

Persistent atrial fibrillation, a large left atrium, low left ventricular ejection fraction, postoperative intravenous pacemaker lead placement through the tricuspid valve, pulmonary hypertension, and severity of preoperative TR have been reported as independent risk factors for postoperative recurrent TR after TAP [4, 5]. Furthermore, there have been reports regarding the relationship between the specific morphological features of tricuspid valve and TR recurrence. The tethering height of the tricuspid valve was reported to have a significant correlation with recurrent TR after TAP [5, 6]. According to the echocardiographic evaluation by Fukuda and colleagues, tethering distances longer than 7.6 mm and tethering area greater than 1.63 cm^2^ were significant predictors of recurrent TR [5]. Furthermore, Yoda et al. reported that a dilated RV (> 40 mm) was significantly associated with recurrent TR after TAP [7]. These findings and the morphological features of the tricuspid valve and RV in the present case (severe TR with extensive tethering height of 12 mm, area of 1.84 mm^2^, and RV end-diastolic dimension of 41 mm) suggested that this patient was an acceptable candidate for TVR to avoid redo tricuspid valve surgery.

In addition to the report by Yoda et al. showing a significant relationship between recurrent TR after TAP and preoperative RV enlargement, Bokma et al. suggested that RV dilation was associated with adverse events after surgery for functional TR and PR with repaired TOF [2]. Thus, the evaluation of RV is considered to be important, and CMR, which is the gold standard for RV evaluation and TAPSE is useful because it is simple and non-invasive [8]. These examinations may provide additional insight into the relationship between recurrent TR after TAP and right ventricular function.

There is controversy regarding the choice of the prosthetic valve in the tricuspid position. A biological valve has the risk of structural degeneration and late reoperation. In contrast, the low pressure and flow in the right side of the heart might induce valve thrombosis in a mechanical prosthesis [9]. Moreover, intravenous ventricular pacemaker lead placement through the mechanical prosthesis at tricuspid position is impossible in patients with bradycardia. Although there are some reports that the long-term outcome and reoperation rate are comparable to mechanical prostheses in tricuspid position [10], controversy still exists about the valvular substitute.

According to literature, Voigt et al. reported a 58-year-old woman who underwent double bioprosthetic valve replacement in right-sided carcinoid heart disease [11]. They proposed that double valve replacement using a bioprosthesis is a reasonable alternative to a mechanical valve, which can avoid life-long anticoagulation therapy with a vitamin K antagonist. Although our patient requires anticoagulation therapy for atrial fibrillation, we proved that right heart double valve replacement using a bioprosthesis is a feasible therapeutic option, even in a middle-aged woman.

Recently, transcatheter interventions for valvular heart diseases, especially aortic valve, have made remarkable progress. Transcatheter tricuspid intervention might be a promising alternative to open surgical procedures for high-risk patients with FTR, including previous cardiac surgery such as the present case, because of its low invasiveness [12]. However, it is still in its infancy, and further investigation of the clinical applicability of the interventions is needed.

## Data Availability

The data are not available for public access due to patient privacy concerns but are available from the corresponding author upon reasonable request.

## Abbreviations

PR: Pulmonary regurgitation
PS: Pulmonary valve stenosis
PVR: Pulmonary valve replacement
RV: Right ventricle
TAP: Tricuspid annuloplasty
TOF: Tetralogy of Fallot
TR: Tricuspid regurgitation
TVR: Tricuspid valve replacement
